# Data to assess the relationship between moral identity and green consumption

**DOI:** 10.1016/j.dib.2018.09.091

**Published:** 2018-10-02

**Authors:** Bo Wu, Zhiyong Yang

**Affiliations:** a Business School, Tianjin University of Finance & Economics, Tianjin, China; b Bryan School of Business and Economics, The University of North Carolina Greensboro, United States

## Abstract

This Data in Brief article is for Study 1 in the manuscript # JEVP-2018-140 (“The Impact of Moral Identity on Consumers’ Green Consumption Tendency: The Role of Perceived Responsibility for Environmental Damage”). It examines the association between moral identity and green consumption. The data was collected using a structured online questionnaire composed of measurements of moral identity, green consumption behavior, social desirability, and demographics (gender, age and financial situation) in China and United States. 259 Chinese and 282 Americans answered the questionnaire. Data was analyzed employing SPSS. Regression analysis was used as statistical tool of analysis.

**Specifications table**

**Table:** 

Subject area	Psychology
More specific subject area	Consumer behavior, green consumption, moral identity
Type of data	Table
How data was acquired	Survey
Data format	Raw
Experimental factors	Sample consisted of online participants in United States and China
Experimental features	Green consumption, moral identity, social desirability and demographics are measured
Data source location	United States and China
Data accessibility	Data is included in this article

**Value of the data**

• This data provides information on green consumption behavior, moral identity, social desirability and demographics across Chinese and American samples.
• The green consumption behavior data can be compared with the sample from other cultures or countries.
• The relationship between moral identity and green consumption behavior can be compared with other cultures or countries.
• The data could be analyzed according to social desirability, gender, age and financial situation.

## 1 Data

The data comprised survey data on green consumption behavior, moral identity, social desirability and demographics. Green consumption refers to the extent to which consumers consider the impact of their own behavior on the environment when they purchase, use, or dispose of products, and try to minimize the negative impact and maximize the positive impact on the environment [1]. Green consumption behavior was measured using Roberts and Bacon׳s [2] 11-item Ecologically Conscious Consumer Behavior scale, which focuses on other-benefit green consumption behavior. Moral identity refers to a structured cognitive schema of moral values, goals, traits and behavioral scripts [3]. It was measured using the five-item Internalization subscale of Aquino and Reed׳s [4] Self-Importance of Moral Identity scale. Reynolds׳s [5] 13-item short form of the Marlowe-Crowne Social Desirability scale was used to measure social desirability. Finally, participants’ gender (0 = *male*; 1 = *female*), age, and financial situation (“*How do you feel about your current financial situation*?” 1 = *extremely bad*; 7 = *extremely good*) were measured. Table 1 shows the partial correlations between study variables.

**Table 1:** Partial correlations between study variables.

Variable	2	3	4	5	6
1. Green Consumption	.268**	.372**	.015	-.149**	.278**
2. Moral Identity	1	.140**	.098*	.134**	.136**
3. Social Desirability		1	.015	-.081	.316**
4. Gender			1	-.089*	.027
5. Age				1	-.006
6. Financial Situation					1

* *p* < .05,

** *p* < .01

Our factor analysis on the combined sample yielded two distinct factors (i.e., moral identity and green consumption behavior), which jointly explained 70.1% of the variance in our data. Both Cronbach׳s alphas of the extracted factors are above the 0.70 threshold. Subsequent analyses on separate samples of Chinese and Americans also confirmed the factor structure and the reliability of the measures.

A regression analysis on green consumption behavior, using moral identity as the independent variable, and social desirability bias, gender, age, and financial situation as covariates, revealed significant coefficients of moral identity (*β*= .23, *t* (535) = 5.94, *p* < .001), social desirability (*β*= .28, *t* (535) = 6.87, *p* < .001), age (*β*= -.16, *t* (535) = -4.13, *p* < .001), and financial situation (*β*= .16, *t* (535) = 3.97, *p* < .001). Gender had no significant effect on green consumption (*p* = .43). Multicollinearity was deemed not a severe threat to the validity of our findings, because all VIFs were lower than 3.3. Separate analyses on the Chinese and the U.S. samples yielded similar results.

## 2 Experimental design, materials and methods

The data presented a quantitative research based on a research design to assess the relationship between moral identity and green consumption [6]. Survey method was deemed suitable for data collection. Two online panels (WJX in China and M-Turk in the U.S.) were selected. 259 Chinese (148 females; *M*_age_ = 32.01, SD = 6.32) and 282 Americans (159 females; *M*_age_ = 37.07, SD = 11.43) took part in this study in exchange for a small monetary compensation. The data was coded and inputted in SPSS version 25. Data was analyzed through regression analysis.
